# Clinical and Echocardiographic Predictors of Major Adverse Cardiovascular Events in ANCA-Associated Vasculitis

**DOI:** 10.3390/medicina62040710

**Published:** 2026-04-08

**Authors:** Ivana Đuran, Bojana Ljubičić, Mira Novković Joldić, Danilo Medin, Milica Knezevic, Nikola Glogonjac, Dragan Vasić, Tijana Azaševac

**Affiliations:** 1 Faculty of Medicine, University of Novi Sad, 21000 Novi Sad, Serbia; bojana.ljubicic@mf.uns.ac.rs (B.L.); mira.novkovic@mf.uns.ac.rs (M.N.J.); danilo.medin@mf.uns.ac.rs (D.M.); 910080d24@mf.uns.ac.rs (M.K.); drvasic09@gmail.com (D.V.); tijana.azasevac@mf.uns.ac.rs (T.A.); 2 Department of Nephrology and Clinical Immunology, University Clinical Centre od Vojvodina, 21000 Novi Sad, Serbia; nikola.glogonjac@kcv.rs; 3 Department of Emergency Internal Medicine, Emergency Centre, University Clinical Centre of Vojvodina, 21000 Novi Sad, Serbia; 4 Clinic of Gastroenterology & Hepatology, University Clinical Centre of Vojvodina, 21000 Novi Sad, Serbia; 5 Clinic for Abdominal, Endocrine and Transplantation Surgery, University Clinical Centre of Vojvodina, 21000 Novi Sad, Serbia

**Keywords:** ANCA-associated vasculitis, cardiovascular risk, echocardiography, valvular heart disease, traditional risk factors

## Abstract

*Background and Objectives*: Cardiovascular disease is the leading cause of mortality in patients with ANCA-associated vasculitis (AAV) after the first year post-diagnosis. This study investigated relationships between traditional risk factors, echocardiographic findings, disease activity, and major adverse cardiovascular events (MACE) in AAV patients. *Aim*: This retrospective single-center study aimed to evaluate the impact of traditional cardiovascular risk factors and echocardiographic parameters on the occurrence of MACE in patients with AAV. *Materials and Methods*: This study included adult patients with AAV who were evaluated between 2020 and 2025. Data collected included demographics, cardiovascular risk factors, Birmingham Vasculitis Activity Score version 3 (BVASv3), laboratory parameters, immunosuppressive therapy, and transthoracic echocardiography (TTE) findings. MACE was defined as myocardial infarction, stroke, revascularization, cardiovascular hospitalization, or cardiovascular death. *Results:* The cohort comprised 32 females (61.5%) and 20 males (38.5%), with a mean age of 62.4 ± 12.4 years. MACE occurred in 38.5% of patients. Male gender (*p* = 0.002), overweight (*p* = 0.016), hyperlipidemia (*p* = 0.003), and prior cardiovascular disease (*p* = 0.002) were significantly associated with MACE in univariate analyses. Patients with MACE had larger left atrial anteroposterior dimensions on the parasternal long-axis view (median 3.9 vs. 3.3 cm, *p* = 0.002) and lower left ventricular ejection fraction assessed by the modified biplane Simpson’s method (median 53% vs. 60%, *p* = 0.002). Valvular dysfunction was not associated with MACE in a statistically significant manner. Disease activity markers (BVASv3 and CRP) showed no significant correlation with cardiovascular events or echocardiographic parameters. *Conclusions*: Our results demonstrate that traditional cardiovascular risk factors are stronger determinants of MACE in AAV patients than disease-specific parameters. Cardiac structural changes, including left atrial dilation and decreased left ventricular ejection fraction, were significantly associated with cardiovascular outcomes. These findings underscore the importance of integrating systematic cardiovascular risk assessment and aggressive risk factor modification into standard AAV management protocols.

## 1. Introduction

ANCA-associated vasculitides comprise a group of systemic autoimmune diseases marked by necrotizing small-vessel inflammation [1]. The spectrum of AAV encompasses granulomatosis with polyangiitis (GPA), microscopic polyangiitis (MPA), and eosinophilic granulomatosis with polyangiitis (EGPA). The pathogenic hallmark of these conditions is the presence of autoantibodies targeting either proteinase 3 (PR3) or myeloperoxidase (MPO), which drive widespread vascular injury and multiorgan dysfunction [2,3]. Although kidney and lung disease are the predominant manifestations, growing evidence suggests that cardiovascular involvement contributes substantially to long-term morbidity and mortality [4,5].

After the initial year post-diagnosis, cardiovascular complications become the principal cause of death in AAV [6,7]. Recent systematic reviews and meta-analyses confirm that patients with AAV face substantially elevated cardiovascular risk, with pooled estimates indicating a 43% increased risk of stroke, 49% increased risk of myocardial infarction, and 79% greater cardiovascular mortality versus matched populations. Population studies reveal that myocardial infarction occurs nearly twice as frequently in AAV, peaking during the initial three months following diagnosis. Notably, cardiovascular risk appears to increase even in the months preceding AAV diagnosis, highlighting the importance of early clinical vigilance and comprehensive risk assessment [8].

Multiple mechanisms drive accelerated atherosclerosis in AAV. Neutrophil activation triggered by ANCA leads to cytokine release and procoagulant factor production, resulting in endothelial injury, premature atherosclerosis, and heightened thrombotic tendency. Recent pathogenetic insights emphasize the role of persistent inflammation and immune dysregulation in the development of cardiovascular complications [9,10]. Long-term immunosuppressive therapy, particularly glucocorticoid exposure, may independently augment cardiovascular risk through metabolic dysregulation and promotion of traditional risk factors [11]. Traditional cardiovascular risk factors, including hypertension, diabetes mellitus, dyslipidemia, smoking, and physical inactivity, are frequently observed in patients with AAV; their independent contribution to cardiovascular outcomes in this population remains incompletely defined. The OSTEOVAS consortium identified age, cardiovascular history, physical inactivity, hypertension, and lipid disorders as independent predictors of MACE [12,13]. Disease severity measured by the Birmingham Vasculitis Activity Score version 3 (BVASv3) also predicts cardiovascular events and related mortality [14].

Subclinical cardiac damage detected by imaging modalities warrants further investigation in AAV. Echocardiography provides a non-invasive assessment of structural and functional heart abnormalities that may precede clinical events. Previous studies have demonstrated that AAV patients frequently exhibit reduced left ventricular systolic function, diastolic dysfunction, and elevated pulmonary arterial pressures compared with controls [15]. Valvular heart disease, though less commonly reported, has been documented in AAV, predominantly affecting patients with GPA and most frequently manifesting as aortic regurgitation (AR) [16]. However, valvular involvement is limited to case reports and small retrospective series, and prospective data on the clinical significance and prognostic implications of valvular dysfunction in AAV are lacking.

We conducted this retrospective single-center study to evaluate the impact of both traditional cardiovascular risk factors and echocardiographic parameters on the occurrence of MACE in a cohort of patients with AAV. Specifically, we sought to determine whether the severity of valvular dysfunction, as quantified by TTE, was associated with MACE in univariate analysis, alongside disease activity, immunosuppressive therapy, and established cardiovascular risk factors. We hypothesized that echocardiographic abnormalities would provide incremental prognostic information and improve cardiovascular risk stratification in this high-risk population.

## 2. Materials and Methods

### 2.1. Study Design

This was a retrospective, observational, single-center study conducted at the Department of Nephrology and Clinical Immunology at the University Clinical Centre of Vojvodina, Novi Sad, Serbia. The institutional ethics committee approved the study protocol, and informed consent was waived due to the retrospective nature of the investigation.

Patients with a confirmed AAV diagnosis who were evaluated and followed at our institution between 2020 and 2025 were eligible for inclusion. Echocardiographic assessment was performed at the time of clinical evaluation or diagnosis, prior to the occurrence of MACE in all included cases. The median follow-up duration was comparable across the cohort. The diagnosis of AAV was established according to internationally recognized classification criteria [17]. Patients fulfilled the American College of Rheumatology (ACR) criteria for GPA and EGPA, and/or the European Medicines Agency (EMA) algorithm, and/or the Chapel Hill Consensus Conference definitions for all AAV subtypes, including MPA.

Inclusion criteria required a confirmed diagnosis of AAV and complete clinical documentation, including laboratory data and TTE findings. Patients with incomplete or missing medical records were excluded from the analysis. A total of 52 patients met the eligibility criteria and were included in the final study cohort.

Demographic, clinical, laboratory, echocardiographic, and treatment-related data were retrospectively extracted from electronic medical records and archived paper charts. Demographic variables included age at the time of echocardiographic examination and sex. Clinical data included the specific AAV subtype (GPA, MPA, EGPA, or ANCA-negative), disease duration when available, and patterns of major organ involvement. Disease activity was quantified using the Birmingham Vasculitis Activity Score version 3 (BVASv3), recorded at the time point closest to the echocardiographic assessment.

Traditional cardiovascular risk factors were systematically documented, including arterial hypertension, diabetes mellitus, dyslipidemia, current or former smoking status, physical inactivity, overweight (defined as body mass index ≥ 25 kg/m^2^), and prior cardiovascular disease [13]. Laboratory parameters included markers of systemic inflammation (C-reactive protein), indices of renal function, and ANCA serological status. Treatment-related variables included cumulative and current use of immunosuppressive agents, particularly cyclophosphamide (CyC).

Echocardiographic data were derived from a single standard TTE performed per patient as part of routine clinical care by experienced operators. Chamber quantification was performed in accordance with the recommendations of the European Association of Cardiovascular Imaging (EACVI) and the American Society of Echocardiography (ASE) [18]. The severity of valvular regurgitation affecting the mitral, aortic, and tricuspid valves was graded on an ordinal scale from 0 to 4 (0 = none, 1 = mild, 2 = moderate, 3 = moderate-to-severe, 4 = severe), in accordance with contemporary echocardiographic guidelines [19,20]. Valvular regurgitation grading was based on a multiparametric qualitative and semi-quantitative approach using the data available in archived echocardiographic reports; quantitative parameters such as effective regurgitant orifice area (EROA), regurgitant volume, and regurgitant fraction were not consistently recorded and could not be systematically extracted. Additional echocardiographic parameters recorded included left ventricular ejection fraction (LVEF), calculated using the modified biplane Simpson’s method (method of discs), left atrial (LA) anteroposterior linear dimension measured in the parasternal long-axis (PLAX) view, and estimated right ventricular systolic pressure (RVSP). RVSP was estimated from peak tricuspid regurgitation velocity using standard Doppler echocardiographic methodology and served as a surrogate marker of pulmonary hypertension. No systematic non-invasive vascular assessment (such as carotid intima-media thickness, coronary artery calcium scoring, or myocardial perfusion imaging) was performed, and myocardial strain analysis (global longitudinal strain) was not available in the archived echocardiographic records. These represent inherent limitations of the retrospective design.

The primary outcome was the occurrence of MACE, defined as a composite endpoint comprising myocardial infarction, ischemic stroke or transient ischemic attack, coronary or peripheral arterial revascularization, hospitalization for cardiovascular causes, or cardiovascular death. MACE occurrence was recorded as a dichotomous variable (yes/no).

Arterial hypertension was defined as a documented diagnosis in the medical record or ongoing antihypertensive pharmacotherapy. Diabetes mellitus was defined as a documented diagnosis or current use of glucose-lowering therapy. Dyslipidemia was defined as a documented diagnosis or treatment with lipid-lowering agents. Smoking status was categorized as current or former smoking based on available documentation. Overweight was defined as body mass index ≥ 25 kg/m^2^ when anthropometric data were available. Previous cardiovascular disease was defined as a documented history of myocardial infarction, stroke, or transient ischemic attack, coronary or peripheral revascularization, or heart failure before echocardiographic evaluation.

ANCA serological status was classified as PR3-ANCA positive, MPO-ANCA positive, or ANCA-negative, using enzyme-linked immunosorbent assay (ELISA) or chemiluminescence immunoassay (CLIA), performed according to standard laboratory protocols and the manufacturer’s instructions. ANCA-negative AAV was defined as patients fulfilling clinical, histopathological, and/or radiological criteria for AAV (according to ACR criteria, EMA algorithm, or Chapel Hill Consensus Conference definitions) in whom both PR3-ANCA and MPO-ANCA were undetectable by standard immunoassay testing at the time of diagnosis and during follow-up. Importantly, ANCA negativity does not exclude the diagnosis of AAV when other diagnostic criteria are met, particularly in patients with EGPA, where ANCA negativity rates may exceed 50% [17].

Disease activity was quantified using the BVASv3, recorded at the time point closest to the echocardiographic study.

### 2.2. Statistical Analysis

Statistical analysis was performed using SPSS version 31.0 (IBM Corp., Armonk, NY, USA). Categorical variables were presented as frequencies and percentages, with differences between groups assessed using the chi-square test. For continuous variables, normality of distribution was first evaluated using the Kolmogorov–Smirnov test, measures of skewness and kurtosis, and homogeneity of variance was assessed using Levene’s test for equality of variances. Continuous variables that met the assumptions for parametric analysis were reported as mean ± standard deviation (SD), and differences between groups were analyzed using an independent *t*-test. For variables with non-normal distribution, central tendency measures were presented as median and interquartile range (IQR), and differences between groups were tested using the Mann–Whitney U test. When comparing three groups of patients (ANCA-negative, MPO, and PR3), differences were analyzed using one-way analysis of variance (ANOVA) or its Non-parametric alternative (Kruskal–Wallis test). The presence of correlations between the examined disease activity variables and the measured echocardiographic parameters was assessed using Spearman’s rank correlation, given that some of the variables included in the analysis deviate from a normal distribution.

A *p*-value < 0.05 was considered statistically significant. All statistical comparisons were univariate in nature; no multivariable regression model was constructed, given the limited sample size and the relatively small number of events, which would not provide adequate statistical power for stable adjusted analyses.

## 3. Results

A total of 52 patients with confirmed ANCA-associated vasculitis were included in the study. The cohort comprised 32 females (61.5%) and 20 males (38.5%), with a mean age of 62.4 ± 12.4 years. MACE occurred in 20 patients (38.5%) during the follow-up period. The distribution of AAV subtypes and baseline characteristics across ANCA serotype groups are presented in Table 1, and the comparison between patients with and without MACE is shown in Table 2.

### 3.1. Patient Characteristics and ANCA Serotype Distribution

The cohort included 14 ANCA-negative patients (26.9%), 18 PR3-ANCA-positive patients (34.6%), and 20 MPO-ANCA-positive patients (38.5%). The mean age was comparable across serotype groups (62.1 ± 14.3, 62.3 ± 10.1, and 62.7 ± 13.5 years, respectively; *p* = 0.991). The proportion of male patients was also similar across groups (28.6%, 44.4%, and 40.0%, respectively; *p* = 0.647). No statistically significant differences were observed between ANCA serotype groups in terms of BMI, smoking status, comorbidities, laboratory parameters, disease activity (BVASv3), or echocardiographic findings (all *p* > 0.05), as detailed in Table 1. The overall prevalence of arterial hypertension was 75.0%, diabetes mellitus 36.5%, and hyperlipidemia 53.8%. Prior cardiovascular disease was present in 48.1% of patients. Median CRP was 11.7 mg/L, and mean BVASv3 score was 19.2 ± 9.4, with no significant differences across ANCA serotypes.

#### 3.1.1. MACE Occurrence and Associated Risk Factors

MACE was recorded in 20 patients (38.5%). Male sex was significantly more prevalent in the MACE group (65.0% vs. 12.3%, *p* = 0.002). Patients with MACE had a significantly higher median BMI compared with those without MACE (25.4 vs. 23.5 kg/m^2^, *p* = 0.016). Hyperlipidemia was present in 80.0% of patients with MACE versus 37.5% of those without MACE (*p* = 0.003). Prior cardiovascular disease was significantly more frequent in the MACE group (75.0% vs. 31.2%, *p* = 0.002). Arterial hypertension was present in 75.0% of the total cohort, but did not differ significantly between groups (80.0% vs. 71.9%, *p* = 0.510). Similarly, diabetes mellitus (45.0% vs. 31.2%, *p* = 0.316) and smoking status (55.0% vs. 31.2%, *p* = 0.089) showed no statistically significant association with MACE occurrence. Platelet count was higher in the MACE group (median 251 vs. 210 × 10^9^/L, *p* = 0.030), while other complete blood count parameters did not differ between groups. Renal function markers (creatinine and urea) and inflammatory parameters (CRP and BVASv3) were comparable between MACE and non-MACE patients (all *p* > 0.05). Detailed comparisons are presented in Table 2.

#### 3.1.2. Echocardiographic Findings and Cardiovascular Outcomes

Significant differences in echocardiographic parameters were observed between patients with and without MACE. Left atrial dimensions were significantly larger in the MACE group compared with the non-MACE group (median 3.9 vs. 3.3 cm, *p* = 0.002). Left ventricular ejection fraction was significantly lower in patients who experienced MACE (median 53% vs. 60%, *p* = 0.002). Right ventricular systolic pressure did not differ significantly between groups (median 35 vs. 31.5 mmHg, *p* = 0.984). Regarding valvular dysfunction, mitral regurgitation grade ≥ 2 was present in 30% of MACE patients and 31.2% of non-MACE patients (*p* = 0.914). Aortic regurgitation grade ≥ 2 was observed in 15.0% of MACE and 12.5% of non-MACE patients (*p* = 0.797). Tricuspid regurgitation grade ≥ 2 was present in 35.0% and 25.0% of MACE and non-MACE patients, respectively (*p* = 0.439). None of the valvular dysfunction parameters were statistically significant for MACE occurrence. A supplementary analysis was performed to evaluate the effect of cyclophosphamide (CyC) therapy on echocardiographic parameters. No statistically significant differences were observed in any echocardiographic parameter—including left atrial dimensions, left ventricular ejection fraction, and right ventricular systolic pressure—between patients who received CyC and those who did not (all *p* > 0.20).

#### 3.1.3. Disease Activity and Inflammatory Markers

Disease activity assessed by BVASv3 and systemic inflammation quantified by CRP were not statistically significantly associated with MACE occurrence. Mean BVASv3 was 21.7 ± 10.1 in the MACE group versus 17.7 ± 8.3 in the non-MACE group (*p* = 0.136). Median CRP was 11.7 mg/L in the MACE group and 11.0 mg/L in the non-MACE group (*p* = 0.118). No significant difference was identified between BVASv3 or CRP and echocardiographic parameters across the study cohort, further supporting the predominant role of traditional cardiovascular risk factors over disease-specific inflammatory activity in determining cardiovascular outcomes in AAV. Spearman correlation analysis revealed no statistically significant correlations between BVASv3 and any of the echocardiographic parameters assessed, including LA dimensions (r = 0.233, *p* = 0.100), LVEF (r = 0.037, *p* = 0.802), and RVSP (r = −0.066, *p* = 0.647). Similarly, CRP did not correlate significantly with LA dimensions (r = 0.149, *p* = 0.300), LVEF (r = −0.160, *p* = 0.276), or RVSP (r = 0.046, *p* = 0.749).

## 4. Discussion

In our study, male gender, overweight, hyperlipidemia, and previous cardiovascular events were significantly more frequent in the MACE group in univariate comparisons (*p* = 0.002, *p* = 0.016, *p* = 0.003, and *p* = 0.002, respectively). These findings are consistent with studies suggesting that traditional risk factors are important correlates of cardiovascular morbidity and mortality [12,21].

In our analysis, echocardiographic parameters showed that AAV patients with MACE had larger LA dimensions (median 3.9 cm vs. 3.3 cm, *p* = 0.002) and lower LVEF (median 53% vs. 60%, *p* = 0.002) than AAV patients without MACE in univariate analyses. These associations should be interpreted cautiously, given the retrospective design and the high prevalence of pre-existing cardiovascular comorbidities in this cohort. The research of Ahn et al. demonstrated that left atrial enlargement is a well-known marker of increased cardiovascular risk and is associated with hypertension, diastolic dysfunction, atrial fibrillation, and increased stroke risk [15].

Mitral regurgitation of grade 2 or higher was observed in 30% of patients with MACE and 31.2% of patients without MACE (*p* = 0.914). None of the valvular lesions showed a statistically significant association with MACE. Notably, 30% of patients with grade 2+ mitral regurgitation experienced MACE. An important limitation of this analysis is that the etiology of mitral regurgitation—primary (degenerative) versus secondary (functional)—was not systematically documented in archived echocardiographic reports and could not be reliably classified for all patients. Secondary mitral regurgitation, resulting from left ventricular remodeling and annular dilatation in the context of ischemic cardiomyopathy, would represent a consequence of MACE rather than a predictor, and its presence in patients with prior cardiovascular disease would be expected. Future prospective studies should systematically classify the etiology of mitral regurgitation in accordance with current guidelines. These observations do not support our initial hypothesis that the severity of valvular dysfunction, as assessed by transthoracic echocardiography, would be associated with MACE after accounting for disease activity, immunosuppressive treatment, and traditional cardiovascular risk factors. Nonetheless, the prevalence of valvular abnormalities in our cohort was substantially higher than historically reported in AAV, suggesting that valvular involvement may be underrecognized in this population. Prior literature has largely consisted of case reports and small retrospective series, primarily describing aortic regurgitation in patients with granulomatosis with polyangiitis [22]. Our findings indicate that mitral regurgitation is at least as common as, and possibly more frequent than, aortic regurgitation in AAV and warrants further study in prospective cohorts with systematic valvular characterization. Clinically, although no statistically significant differences in MACE occurrence were observed according to the presence or severity of valvular dysfunction in this cohort, the high prevalence of valvular abnormalities supports routine echocardiographic screening in patients with AAV. Larger, prospective studies with extended follow-up, serial echocardiographic evaluations, and more detailed mechanistic characterization are required to clarify the potential prognostic relevance of valvular involvement in AAV.

One of the most significant implications of our study is the absence of association between disease activity parameters (BVASv3 score and CRP) and cardiovascular events. Spearman correlation analysis did not show statistically significant correlations between BVASv3 or CRP and any of the echocardiographic parameters (LA, LVEF, RVSP), with *p*-values greater than 0.10 for all analyzed associations. It should be noted that both BVASv3 and CRP were recorded at a single time point closest to the echocardiographic assessment and do not reflect cumulative inflammatory burden over the disease course. Serial measurement of these parameters over time—which would more accurately capture the longitudinal relationship between inflammation and cardiovascular risk—was not feasible in this retrospective design. This represents an important limitation when interpreting the absence of association, as a single cross-sectional assessment may not adequately represent the patient’s overall inflammatory trajectory. Nonetheless, our findings are consistent with the study of Wallace et al. [6] and suggest that traditional cardiovascular risk factors may exert a dominant influence on MACE independently of contemporaneous disease activity markers.

The relationship between ANCA antibody serotype and right ventricular systolic pressure represented an exploratory observation in our study. Kruskal–Wallis analysis did not reach statistical significance (*p* = 0.055), and the observed pattern of lower RVSP values in MPO-ANCA positive patients [median 29 mmHg (IQR 27–32)] compared to PR3-ANCA positive [32 mmHg (IQR 29–37)] and ANCA-negative patients [38 mmHg (IQR 35–43)] should be interpreted with caution given the limited sample size and the absence of statistical significance. Recent research [23,24] has shown that although ANCA antibody type affects clinical manifestations and patterns of organ involvement, it is not an independent predictor of cardiovascular events.

We also analyzed the effect of CyC therapy on echocardiographic parameters. The results showed no statistically significant differences in any echocardiographic parameter between patients receiving CyC and those not receiving the drug (all *p* > 0.20). CyC cardiotoxicity is well known, especially at oncological doses, but at AAV doses (cumulative dose usually <10–15 g), cardiotoxic effects are rare. Our results are consistent with two studies [4,25] that found no increased risk of cardiovascular events associated with the cyclophosphamide dose used in AAV treatment. However, it should be noted that rituximab is increasingly replacing CyC as first-line therapy for inducing remission in AAV, according to the latest 2025 British Society for Rheumatology (BSR) recommendations. Rituximab may have indirect cardioprotective effects through improved endothelial function, despite transiently increasing cholesterol and triglyceride levels [1,26].

A noteworthy finding in our study was the significantly higher platelet count in patients who experienced MACE compared to those without MACE (median 251 vs. 210 × 10^9^/L, *p* = 0.030). Elevated platelet counts within the upper limits of normal have been previously associated with increased cardiovascular disease risk in the general population, with a platelet count of 301–450 × 10^9^/L associated with a 32% higher risk of cardiovascular disease compared to the reference interval of 201–250 × 10^9^/L [27]. In the context of AAV, ANCA-mediated neutrophil activation generates thrombin via coagulation system stimulation, which subsequently activates platelets through the thrombin-protease-activated receptor (PAR) pathway; activated platelets further amplify the inflammatory cascade by triggering the alternative complement pathway [28]. Furthermore, elevated platelet counts have been demonstrated to serve as a biomarker of disease activity in AAV, correlating with BVASv3 and distinguishing active disease from remission [29]. Of particular clinical relevance, a population-based study from Sweden demonstrated that a high platelet count at the time of AAV diagnosis was associated with an increased risk of stroke [30]. From a mechanistic standpoint, Floyd et al. [21] highlighted that inflammation-driven endothelial dysfunction leads to platelet activation, thereby contributing to a hypercoagulable, prothrombotic state in AAV. Although thrombocytosis in our cohort was not severe (median values remained within or near the upper normal range), the statistically significant difference between MACE and non-MACE groups suggests that platelet count may serve as an integrative biomarker of the underlying prothrombotic and inflammatory state in AAV—and may be more sensitive than single time-point CRP or BVASv3 assessments in reflecting the cumulative cardiovascular risk. This finding is consistent with the interpretation that higher inflammatory activity is indirectly associated with higher cardiovascular risk in AAV, even when contemporaneous acute-phase markers do not reach statistical significance.

## 5. Limitations

Several limitations of this study should be acknowledged. First, the retrospective, single-center design and limited cohort size restrict the generalizability of the findings. Second, the relatively small number of MACE precludes multivariable adjustment; accordingly, all reported associations are based on univariate analyses, and independent predictive value cannot be established. Third, the clinical and serological heterogeneity of the AAV cohort—encompassing GPA, MPA, EGPA, and ANCA-negative patients—may limit the applicability of findings to individual AAV subtypes. Fourth, the possible influence of baseline cardiovascular comorbidity on observed echocardiographic parameters cannot be excluded; in particular, left atrial enlargement and reduced LVEF may reflect pre-existing cardiac remodeling due to prior cardiovascular disease rather than vasculitis-related changes. Fifth, no non-invasive or invasive vascular assessments were performed, and myocardial strain analysis was not systematically available, limiting the ability to attribute findings to specific ischemic or vascular mechanisms. Sixth, disease activity (BVASv3) and inflammatory markers (CRP) were recorded at a single time point and do not reflect cumulative inflammatory burden over the disease course. Seventh, valvular regurgitation grading was based on semi-quantitative methods available in archived reports; quantitative parameters such as EROA, regurgitant volume, and regurgitant fraction were not consistently available, and the etiology of mitral regurgitation (primary vs. secondary) could not be systematically classified. Finally, left atrial size was assessed using linear anteroposterior dimension rather than indexed left atrial volume, which is the guideline-recommended measure for left atrial enlargement.

## 6. Conclusions

The results of this study demonstrate that major adverse cardiovascular events (MACE) in patients with ANCA-associated vasculitis (AAV) are primarily associated with traditional cardiovascular risk factors rather than parameters related to vasculitis itself, its activity, or specific ANCA antibody type. This observation has significant clinical implications and underscores the need for a holistic approach to managing these patients, with particular emphasis on preventing cardiovascular complications. Our findings are in line with accumulating evidence that, despite the elevated cardiovascular risk in AAV, traditional risk factors are important correlates of cardiovascular outcomes, whereas the contribution of disease activity and inflammation remains uncertain.

## Figures and Tables

**Table 1:** Baseline clinical, laboratory, and echocardiographic characteristics of AAV patients stratified by ANCA serotype.

			ANCA-NEG	PR3-ANCA	MPO-ANCA	Sum	*p*
	Age	Mean ± SD	62.1 ± 14.3	62.3 ± 10.1	62.7 ± 13.5	62.4 ± 12.4	0.991 ^a^
	BMI	Mean ± SD	23.5 ± 2.1	25.7 ± 4.0	25.1 ± 3.0	24.9 ± 3.3	0.150 ^a^
	Sex Male	N (%)	4 (28.6%)	8 (44.4%)	8 (40%)	20 (38.5%)	0.647 ^b^
	Smoker	N (%)	6 (42.9%)	10 (55.6%)	5 (25%)	21 (40.4%)	0.155 ^b^
	Urea (mmol/L)	Median (IQR)	8.2 (5.9)	12 (20.9)	7.7 (9.5)	8.9 (10.4)	0.403 ^c^
	Creatinine (umol/L)	Median (IQR)	92.5 (97.2)	122.5 (190.3)	115.5 (222.3)	119.0 (161.0)	0.114 ^c^
	BVASv3	Mean ± SD	15.8 ± 7.5	23.0 ± 11.4	18.2 ± 7.7	19.2 ± 9.4	0.080 ^a^
	CRP (mg/L)	Median (IQR)	11.0 (20.0)	11.7 (29.9)	11.5 (20.9)	11.7 (28.0)	0.367 ^c^
	Cyclophosphamide	N (%)	3 (21.4%)	8 (44.4%)	12 (60.0%)	23 (44.2%)	0.083 ^b^
**Comorbidities**	Diabetes mellitus	N (%)	6 (42.9%)	8 (44.4%)	5 (25.0%)	19 (36.5%)	0.392 ^b^
	Arterial hypertension	N (%)	11 (78.6%)	15 (83.3%)	13 (65%)	39 (75%)	0.401 ^b^
	Hyperlipidemia	N (%)	6 (42.9%)	12 (66.7%)	10 (50.0%)	28 (53.8%)	0.370 ^b^
	Cardiovascular disease	N (%)	8 (57.1%)	8 (44.4%)	9 (45.0%)	25 (48.1%)	0.729 ^b^
**Complete Blood Count**	White blood cells (×10^9^/L)	Mean ± SD	10.4 ± 5.1	10.7 ± 5.5	9.6 ± 3.0	10.2 ± 4.5	0.760 ^a^
	Neutrophils (×10^9^/L)	Mean ± SD	7.7 ± 5.2	7.8 ± 5.0	7.1 ± 2.7	7.5 ± 4.2	0.862 ^a^
	Lymphocytes (×10^9^/L)	Mean ± SD	1.9 ± 1.0	1.9 ± 1.5	1.5 ± 0.6	1.8 ± 1.1	0.344 ^a^
	Red Blood Cells (×10^12^/L)	Mean ± SD	3.9 ± 0.7	4.0 ± 0.5	4.1 ± 0.5	4.0 ± 0.6	0.513 ^a^
	Hemoglobin (g/L)	Mean ± SD	119.1 ± 20.4	119.3 ± 18.6	123.1 ± 17.3	120.7 ± 18.3	0.766 ^a^
	Platelets (×10^9^/L)	Mean ± SD	247.5 ± 81.7	256.0 ± 102.2	240.1 ± 92.9	247.6 ± 91.9	0.872 ^a^
**Echocardiographic**	Left atrial dimensions (cm)	Mean ± SD	3.6 ± 0.4	3.6 ± 0.7	3.4 ± 0.6	3.5 ± 0.6	0.706 ^a^
	LVEF (%)	Median (IQR)	50.5 (8)	58.5 (7)	59.5 (4.2)	59 (8.2)	0.528 ^c^
	RVSP (mmHg)	Median (IQR)	38 (6.7)	32 (8)	29.5 (4.5)	32 (11)	0.055 ^c^
	Mitral regurgitation 2+	N (%)	6 (42.9%)	6 (33.3%)	4 (20%)	16 (30.8%)	0.349 ^b^
	Aortic regurgitation 2+	N (%)	2 (14.3%)	5 (27.8%)	0 (0%)	7 (13.5%)	0.043 ^b^
	Tricuspid regurgitation 2+	N (%)	6 (42.9%)	6 (33.3%)	3 (15.0%)	15 (28.9%)	0.184 ^b^
**Sum**			14 (100%)	18 (100%)	20 (100%)	52 (100%)	

**Legend:** BMI—Body Mass Index, BVASv3—Birmingham Vasculitis Activity score version 3, CRP—C-Reactive Protein, LVEF—Left Ventricular Ejection Fraction, RVSP—Right Ventricular Systolic Pressure; **Statistical tests:** ^a^ ANOVA, ^b^ Chi square test, ^c^ Kruskal–Wallis test.

**Table 2:** Comparison of clinical, laboratory, and echocardiographic characteristics between AAV patients with and without major adverse cardiovascular events (MACE).

			No MACE	MACE	Sum	*p*
	Age	Median (IQR)	62.5 (12.2)	65.0 (9.5)	64.0 (12.0)	0.376 ^a^
	BMI	Median (IQR)	23.5 (2.4)	25.4 (3.7)	24.0 (3.5)	0.016 ^a^
	Sex Male	N (%)	7 (12.3%)	13 (65.0%)	20 (38.5%)	0.002 ^b^
	Smoker	N (%)	10 (31.2%)	11 (55.0%)	21 (40.4%)	0.089 ^b^
	Urea (mmol/L)	Median (IQR)	8.6 (10.7)	9.0 (9.5)	8.9 (10.4)	0.658 ^a^
	Creatinine (umol/L)	Median (IQR)	114.0 (135.5)	120.0 (145.0)	118.0 (161.0)	0.402 ^a^
	BVASv3	Mean ± SD	17.7 ± 8.3	21.7 ± 10.1	19.2 ± 9.4	0.136 ^c^
	CRP (mg/L)	Median (IQR)	11 (19.9)	11.7 (32.4)	11.7 (28.0)	0.118 ^a^
**Comorbidities**	Diabetes mellitus	N (%)	10 (31.2%)	9 (45.0%)	19 (36.5%)	0.316 ^b^
	Arterial hypertension	N (%)	23 (71.9%)	16 (80.0%)	39 (75.0%)	0.510 ^b^
	Hyperlipidemia	N (%)	12 (37.5%)	16 (80.0%)	28 (53.8%)	0.003 ^b^
	Cardiovascular disease	N (%)	10 (31.2%)	15 (75.0%)	25 (48.1%)	0.002 ^b^
**Complete Blood Count**	White blood cells (×10^9^/L)	Median (IQR)	8.7 (4.5)	9.1 (4.8)	8.9 (4.6)	0.756 ^a^
	Neutrophils (×10^9^/L)	Median (IQR)	5.8 (4.3)	7.1 (3.6)	6.8 (4.2)	0.700 ^a^
	Lymphocytes (×10^9^/L)	Median (IQR)	1.5 (1.1)	1.7 (1.2)	1.5 (1.2)	0.785 ^a^
	Red Blood Cells (×10^12^/L)	Median (IQR)	4.2 (0.9)	4.2 (0.9)	4.2 (0.9)	0.326 ^a^
	Hemoglobin (g/L)	Median (IQR)	122 (22.2)	123 (33.5)	122.5 (22.5)	0.821 ^a^
	Platelets (×10^9^/L)	Median (IQR)	210 (87.2)	251 (129.5)	226.0 (113.8)	0.030 ^a^
**Echocardiographic**	Left atrial dimensions (cm)	Median (IQR)	3.3 (0.6)	3.9 (0.5)	3.5 (0.9)	0.002 ^a^
	LVEF (%)	Median (IQR)	60.0 (4.0)	53 (11.2)	59.0 (8.2)	0.002 ^a^
	RVSP (mmHg)	Median (IQR)	31.5 (11.0)	35 (9.5)	32.0 (11.0)	0.984 ^a^
	Mitral regurgitation 2+	N (%)	10 (31.2%)	6 (30%)	16 (30.8%)	0.914 ^b^
	Aortic regurgitation 2+	N (%)	4 (12.5%)	3 (15.0%)	7 (13.5%)	0.797 ^b^
	Tricuspid regurgitation 2+	N (%)	8 (25.0%)	7 (35.0%)	15 (28.8%)	0.439 ^b^

**Legend:** MACE—Major Adverse Cardiovascular Events, BMI—Body Mass Index, BVASv3—Birmingham Vasculitis Activity score version 3, CRP—C-Reactive Protein, LVEF—Left Ventricular Ejection Fraction, RVSP—Right Ventricular Systolic Pressure; **Statistical tests:** ^a^ ANOVA, ^b^ Chi square test, ^c^ Kruskal–Wallis test.

## Data Availability

The data presented in this study are available upon request from the corresponding author. The data are not publicly available due to them containing information that could compromise the privacy of research participants.

## Abbreviations

The following abbreviations are used in this manuscript:

AAV	ANCA-associated vasculitis
ACR	American College of Rheumatology
ANCA	Anti-neutrophil cytoplasmic antibody
AR	Aortic regurgitation
BMI	Body mass index
BSR	British Society for Rheumatology
BVASv3	Birmingham Vasculitis Activity Score version 3
CLIA	Chemiluminescence immunoassay
CRP	C-reactive protein
EGPA	Eosinophilic granulomatosis with polyangiitis
ELISA	Enzyme-linked immunosorbent assay
EMA	European Medicines Agency
GPA	Granulomatosis with polyangiitis
IQR	Interquartile range
LA	Left atrium/left atrial
LVEF	Left ventricular ejection fraction
MACE	Major adverse cardiovascular events
MPA	Microscopic polyangiitis
MPO	Myeloperoxidase
PAR	Protease-activated receptor
PR3	Proteinase 3
RVSP	Right ventricular systolic pressure
SD	Standard deviation
TTE	Transthoracic echocardiography
